# Urine flow rate monitoring in hypovolemic multiple trauma patients

**DOI:** 10.1186/s13017-017-0152-3

**Published:** 2017-08-18

**Authors:** Evgeni Brotfain, Yoram Klein, Ronen Toledano, Leonid Koyfman, Dmitry Frank, Micha Y. Shamir, Moti Klein

**Affiliations:** 1 Trauma unit, Sheba Medical Center, Tel Aviv University, Tel Aviv, Israel; 2 Department of Anesthesiology and Critical Care, General Intensive Care Unit, Soroka Medical Center, Ben-Gurion University of the Negev, Beer Sheva, Israel; 3 Clinical Research Center, Soroka Medical Center, Ben-Gurion University of the Negev, Beer Sheva, Israel; 4 Department of Anesthesiology, Hadassah–Hebrew University Medical Center, Jerusalem, Israel

**Keywords:** Minute-to-minute urine flow rate, Urine flow rate variability, Monitoring, Multiple trauma

## Abstract

**Background:**

The urine output is an important clinical parameter of renal function and blood volume status, especially in critically ill multiple trauma patients. In the present study, the minute-to-minute urine flow rate and its variability were analyzed in hypotensive multiple trauma patients during the first 6 h of their ICU (intensive care unit) stay. These parameters have not been previously reported.

**Methods:**

The study was retrospective and observational. Demographic and clinical data were extracted from the computerized Register Information Systems. A total of 59 patients were included in the study. The patients were divided into two study groups. Group 1 consisted of 29 multiple trauma patients whose systolic blood pressure was greater than 90 mmHg on admission to the ICU and who were consequently deemed to be hemodynamically compromised. Group 2 consisted of 30 patients whose systolic blood pressure was less than 90 mmHg on admission to the ICU and who were therefore regarded as hemodynamically uncompromised.

**Results:**

The urine output and urine flow rate variability during the first 6 h of the patients’ ICU stay was significantly lower in group 2 than in group 1 (*p* < 0.001 and 0.006 respectively). Statistical analysis by the Pearson method demonstrated a strong direct correlation between decreased urine flow rate variability and decreased urine output per hour (*R* = 0.17; *P* = 0.009), decreased mean arterial blood pressure (*R* = 0.24; *p* = 0.001), and increased heart rate (*R* = 0.205; *p* = 0.001).

**Conclusion:**

These findings suggest that minute-to-minute urine flow rate variability is a reliable incipient marker of hypovolemia and that it should therefore take its place among the parameters used to monitor the hemodynamic status of critically ill multiple trauma patients.

## Background

Trauma-associated acute hemorrhage is a leading cause of intravascular volume depletion [1–3] ranging from mild hypovolemia to hemorrhagic shock [1, 2]. The foremost priorities in acute trauma are hemorrhage control and hemodynamic resuscitation [1]. The urine output is a vital clinical parameter of renal function and blood volume status, especially in critically ill multiple trauma patients during their hospital admission and ICU (intensive care unit) stay [3, 4]. It is typically measured hourly and expressed in milliliters per hour. However, the blood volume status and the renal function of multiple trauma patients change more rapidly, especially during the first 24 h of ICU admission [5]. In previously published animal and human studies [6, 7], the use of a continuous minute-to-minute urine flow rate monitoring system (URINFO™, FlowSense Medical, Misgav, Israel) has been shown to detect hypovolemia earlier than other standard parameters. The minute-to-minute UFR was found to be a dynamic variable, which significantly decreases during acute bleeding and is restored after rehydration [6]. Furthermore, an animal model of the minute-to-minute UFR demonstrated that during euvolemia there is variation in the flow rate and that this parameter decreases and eventually disappears during acute hemorrhage. This variability has also been shown to decrease and eventually disappear during acute gradual hemorrhage [7].

This study analyzed minute-to-minute UFR and its variability in hypotensive multiple trauma patients during the first 6 h of their admission to the intensive care unit (ICU). This is the first such study of the significance of these parameters.

## Methods

Soroka Medical Center is a University Level I trauma center with approximately 2500–3000 trauma admissions per year. About 10% of those trauma patients have an Injury Severity Score (ISS) of 16 or above (severe trauma). The study was retrospective and observational. Clinical and laboratory data were collected retrospectively from the records of all multiple trauma patients hospitalized in the Soroka Medical Center general intensive care unit (GICU) between January 2013 and January 2014. All the clinical data were extracted from the computerized Register Information Systems (MetaVision® and iMDsoft®, Israel). The Human Research and Ethics Committee at Soroka Medical Center in Beer Sheva, Israel, approved this study (RN-SOR-0158-14). The patients’ concern has not been needed because of retrospective nature of the study. Patients were not involved in the design or recruitment of the study.

### Inclusion criteria

All multiple trauma patients over the age of 18 who were admitted to the GICU for more than 24 h were considered eligible for inclusion in the study.

### Exclusion criteria

Patients who stayed in the GICU for less than 24 h were excluded from the study. Also, anuric patients on admission to the GICU as well as patients previously known to have chronic renal failure or kidney disease were excluded from the study. Lastly, patients were excluded if their medical record data were incomplete.

### Variables and measurements

The following data were collected: demographic data (age, gender, weight); minute-to-minute UFR (see below; urine output per hour; total fluid balance per hour; heart rate; arterial blood pressure; body temperature; central venous pressure (CVP); arterial blood pH and lactate and bicarbonate levels, serum urea, creatinine, sodium and chloride levels; hemoglobin level; admission trauma diagnosis; and the APACHE-II score on admission to the ICU. Clinical data was collected for the first 6 h of ICU stay. Data on significant therapeutic measures (administration of intravenous fluids, blood products, and vasopressors) and clinical parameters (vital signs) were collected for the first 6 h of the patients’ ICU stay.

### Scores

Severity of critical illness and multiorgan failure were evaluated by the APACHE II (Acute Physiology and Chronic Health Evaluation II) score within 24 h of admission to the GICU.

### URINFO2000™ (FlowSense Medical, Misgav, Israel)

All multiple trauma patients admitted to the GICU underwent insertion of a Foley catheter which was routinely connected to a URINFO 2000™ device. URINFO 2000™ (FlowSense Medical, Misgav, Israel) is a novel urine collecting and urine flow measurement system that uses an optical drop detector to measure urine flow every 3 min through a measuring chamber. The detector enables the reliable calculation of the UFR at varying flow rates and urine osmolarities. The system was connected to the computerized patients’ record system of the GICU.

### UFR variability

The minute-to-minute UFR variability was defined and calculated as the variance of UFR changes from minute to minute [7]. The URINFO 2000™ (Flow Sense Medical, Misgav, Israel) is a novel urine collecting and urine flow measurement system that uses an optical drop detector to measure urine flow every 3 min through a measuring chamber. The difference between two consecutive values (total 6 min), divided by the first value, multiplied by 100. The result of the formula represents the percentage of change from the measured value every 3 min.

### Study groups

The patients were divided into two study groups. Group 1 consisted of 29 multiple trauma patients and who were consequently deemed hemodynamically stable on admission to ICU (systolic blood pressure (SBP) was greater than 90 mmHg, mean arterial pressure more than 65 mmHg, central venous pressure more than 8 cmH_2_O) on admission to the ICU. Group 2 consisted of 30 patients and who regarded as hemodynamically unstable (SBP was less than 90 mmHg, mean arterial pressure less than 65 mmHg, central venous pressure less than 8 cmH_2_O, elevated blood lactate level) at admission to the ICU.

### Statistical analysis

The two study groups (“compromised” and “uncompromised”) were compared for UFR variability which was the primary parameter under study.

The patients’ characteristics and outcomes were compared using chi-square or Fisher’s exact tests for categorical variables. Continuous variables were analyzed with a Student’s *t* test or the Mann-Whitney Test, depending on the validity of the normality assumption.

For comparison of minute-to-minute urine rate variability, the coefficient of variation was calculated and analyzed with a Student’s *t* test. The Pearson method was used to analyze statistical correlation between different vital parameters.

Dynamic changes of UFR variability during the first 6 h of ICU stay were demonstrated by the LOESS curves non-parametric regression method. A *p* value of less than 0.05 represents a statistically significant finding. Statistical analyses were performed using IBM SPSS Statistics 20 (IBM Corp.).

## Results

Initially, the clinical and laboratory data of 120 critically ill multiple trauma patients admitted to the ICU during the study period were analyzed. Of these, 59 patients were eventually included in the study (Table 1).The remaining 61 patients were excluded on the basis of the exclusion criteria (of them, 30 patients were oligo-anuric on admission to ICU have chronic renal failure or kidney disease or had previously documented chronic renal failure or kidney disease, 9 patients had incomplete medical record data and 22 patients were hospitalized in our ICU less than 24 h). The patients’ epidemiological and clinical characteristics are summarized in Table 1. The two groups (“uncompromised” and “compromised”) were similar in age, gender, weight, APACHE score, and length of ICU stay.

**Table 1:** Patients’ demographic data, clinical outcome and underlying condition

	Group 1 (*n* = 29)	Group 2 (*n* = 30)	*p* value (95%CI)*
Age, years (mean ± SD)	35.34 ± 11.71	36.66 ± 13.27	0.68
Male gender (%)	27 (93.1)	26 (86.7)	0.67
Weight, kg (mean ± SD)	76.65 ± 14.75	70.4 ± 12.32	0.72
APACHE II, units (mean ± SD)	24 (24–27.5)	24.5 (22–27.25)	0.41
ICU stay, days (mean ± SD)	7.3 ± 2.9	7.91 ± 1.8	0.2

*P values less than 0.05 considered to be statistically significant

The patients in group 2 had significantly lower systemic and mean arterial pressures and higher heart rates on admission to the ICU compared to the patients in group 1 (*p* < 0.001, Table 2).

**Table 2:** Vital signs (mean ± SD)

	Group 1 (*n* = 29)	Group 2 (*n* = 30)	*p* value (95%CI)*
Systolic blood pressure (SBP) (mmHg)^a^	121.58 ± 21.2	82.6 ± 5.28	< 0.001
Mean arterial blood pressure (MAP) (mmHg)^a^	81.13 ± 16.32	55.33 ± 5.59	< 0.001
Heart rate (beat/min)^a^	100.78 ± 1.80	118.86 ± 23.71	< 0.001
Urine output per hour (ml)^b^	151.75 ± 29.22	63.58 ± 3.04	< 0.001
Urine flow rate variability (mean, ml)^b^	4.29 ± 1.3	3.78 ± 1.46	0.006

*P values less than 0.05 considered to be statistically significant

^a^Vital signs at admission to ICU (mean ± SD)

^b^Mean of urine output and urine flow rate variability per hour during first 6 h of ICU stay

The UO and the UFR variability during the first 6 h of the patients’ ICU stay were significantly lower in the group 2 patients than in the group 1 patients (*p* < 0.001 and 0.006 respectively).

The CVP and arterial blood pH were also significantly lower in the group 2 patients than in the group 1 patients (*p* < 0.001; Table 3). The laboratory parameters and body temperatures of the patients in both groups were similar during the first 6 h of their ICU stay (Table 3).

**Table 3:** Clinical and laboratory data of study group patients (mean ± SD)

	Group 1 (*n* = 29)	Group 2 (*n* = 30)	*p* value (95%CI)*
Hemoglobin (g\dL)	11.91 ± 2.04	11.36 ± 2.0	0.31
Serum sodium (mmol/L)	137.03 ± 3.26	137.93 ± 2.56	0.24
Serum chloride (mmol/L)	106.2 ± 4.44	106.56 ± 4.51	0.76
Arterial blood pH	7.33 ± 0.05	7.24 ± 0.07	< 0.001*
Arterial blood bicarbonate (mmol/L)	22.45 ± 2.31	20.54 ± 2.31	0.002
Arterial blood lactate (mmol/L)	1.77 ± 0.88	2.41 ± 1.06	0.015
CVP (mmHg)	10.03 ± 1.76	2.26 ± 1.59	< 0.001
Body temperature (°C)	36.63 ± 0.63	35.53 ± 0.82	0.09

*P values less than 0.05 considered to be statistically significant

Statistical analysis by the Pearson method demonstrated strong direct correlation between decreased UFR variability and the following parameters: decreased urine output per hour (*R* = 0.17; *p* = 0.009); decreased mean arterial blood pressure (MAP) (*R* = 0.24, *p* = 0.001); and increased heart rate (*R* = 0.205, *p* = 0.001). No correlation with systolic blood pressure (*R* = 0.11, *p* = 0.073).

**Fig. 1:**
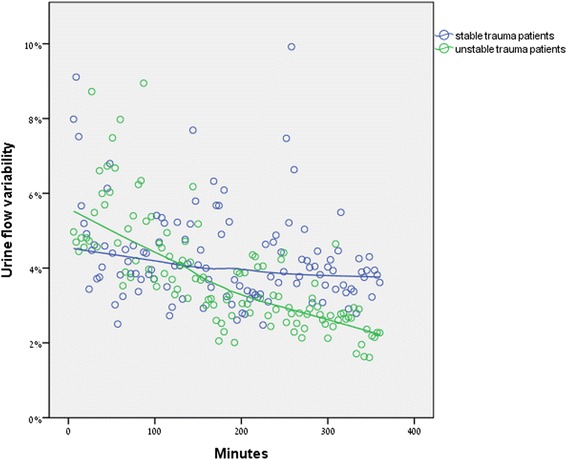
Dynamic changes in urine flow rate variability during the first 6 h of ICU stay. (Variability and time) (Note: blue—group 1 [compromised trauma patients]; green—group 2 [uncompromised trauma patients])

The dynamic changes in UFR variability are graphically presented in Fig. 1. In contrast to the wide UFR variability found in the group 1 (uncompromised) patients, we demonstrated a significant trend towards decreased UFR variability in the group 2 (compromised) patients during the first 6 h of their ICU stay (Fig. 1).

## Discussion

The American College of Surgeons Committee on Trauma guidelines define hypotension as a systolic blood pressure (SBP) of less than 90 mmHg [1]. This threshold is often used as a marker of hemodynamic instability in adult patients with multiple trauma [8–10]. The cornerstone of appropriate management of multiple trauma patients is the early evaluation of their hemodynamic status to detect potential hemorrhage or subacute (“occult”) hypoperfusion [11–13]. The initial assessment of these patients and decisions regarding further administration of resuscitatory fluid or blood products are accomplished by monitoring the patients’ vital signs, especially blood pressure, heart rate, and urine output. However, there are cases in which the clinical picture is difficult to interpret because of the presence of pain, hyper/hypothermia, neurogenic or cardiogenic shock, or other factors [14]. An ideal monitoring device for such patients would be noninvasive, small in size, transportable, and easy to use and understand. It should be able to provide early assessment of even a very minor degree of hypoperfusion and as well as information on the adequacy of blood volume resuscitation [15].

Optimal and adequate treatment of hypoperfusion is crucial in preventing the development of reperfusion injury, the systemic inflammatory response syndrome (SIRS) and further progression to irreversible multiple organ failure [11]. Despite “normalization of vital signs”, including maintenance of a urine output of 0.5–1 cm^3^/kg/h, occult hypoperfusion can still be present in multiple trauma patients [11–13]. In the last two decades, a large variety of noninvasive continuous hemodynamic monitoring devices have been introduced into trauma centers worldwide. However, UO is the single important clinical parameter that is not monitored electronically in most centers. Early recognition of renal dysfunction in multiple trauma patients is extremely important in the prevention of further kidney injury, a complication which often adversely affects the clinical outcome [16]. In recent years, Urinfo, a new digital continuous minute-to-minute UFR monitoring device (URINFO™, FlowSense Medical, Misgav, Israel) has been shown to be significantly superior to manual nurse-handled urinometers in terms of accuracy of measurement, ease of handling, and staff satisfaction [4].

In a previous study, Shamir et al. [6] described the successful use of continuous minute-to-minute UFR monitoring in 11 patients who underwent elective spine surgery. They demonstrated that the kidneys show rhythmic variation in the minute-to-minute UFR, a phenomenon which is probably mediated by pacemaker activity located in the proximal portion of the upper urinary tract and influenced by prostaglandins and sensory nerves [17]. A decrease in the variability of the minute-to-minute UFR was an early sign of bleeding or hypovolemia in these previously healthy patients during their elective spinal surgical procedures [6]. Moreover, these authors [6] showed that the UFR variability not only decreased during the bleeding process but also returned to baseline after rehydration. In animal models, Klein et al. [7] demonstrated a strong correlation between the decrease in URF variability and the onset of hypovolemia induced by controlled bleeding.

In the present study, we analyzed clinical data describing minute-to-minute UFR and urine flow variability in multiple trauma patients. We found a significant decrease in minute-to-minute UFR and urine flow variability in multiple trauma patients who presented to the ICU with hemodynamic compromise (SBP less than 90 mmHg) compared to trauma patients who were hemodynamically normal. In both study groups, minute-to-minute UFR and urine flow variability decreased during the first 6 h of ICU admission in parallel with decreases in SBP and MAP and increases in heart rate and arterial blood lactate levels. Importantly, trauma patients in both study groups had an “adequate” (about 1 cm^3^/kg/h) urine output. We found a strong clinical correlation between decreased UFR variability and decreased urine output per hour, decreased mean arterial pressure and increased heart rate in the group 2 (unstable) trauma patients. Thus, the minute-to-minute UFR/urine flow variability as a continuous, sensitive measurement has a significant clinical advantage and superior to other vital parameters as an early diagnosis of hypovolemia in multiple trauma patients.

Our study has a number of limitations. The main limitations are its retrospective design and the small number of patients included in the study. Also, there is no sample size calculation. Furthermore, because our study is retrospective, the influence of directed, active control of SBP on the minute-to-minute UFR, and urine flow variability could not be estimated or taken into account.

Finally, it seems that low minute-to-minute UFR variability correlate with hypovolemia and that means it occur in opposite. Future investigations in a new prospective design setup with control of all resuscitative parameters need to be provided to clarify that.

## Conclusions

We suggest that UFR variability can serve as a reliable incipient marker of occult hypovolemia and also as an indicator of the end-point of blood volume resuscitation. We therefore propose that it should be one of the parameters used to monitor the hemodynamic status of critically ill multiple trauma patients. Furthermore, in view of our findings, we suggest that more comprehensive randomized and prospective studies should be undertaken to evaluate the potential clinical role of UFR variability and its influence on the ICU outcome of multiple trauma patients.

## Abbreviation

UFR: Urine flow rate
